# The microenvironment—a general hypothesis on the homeostatic function of extracellular vesicles

**DOI:** 10.1096/fba.2021-00155

**Published:** 2022-03-12

**Authors:** Amber N. Stratman, Clair Crewe, Philip D. Stahl

**Affiliations:** ^1^ Department of Cell Biology and Physiology Washington University School of Medicine St. Louis Missouri USA; ^2^ Department of Internal Medicine Division of Endocrinology, Metabolism and Lipid Research Washington University School of Medicine St. Louis Missouri USA

## Abstract

Extracellular vesicles (EVs), exosomes and microvesicles, is a burgeoning field of biological and biomedical research that may change our understanding of cell communication in plants and animals while holding great promise for the diagnosis of disease and the development of therapeutics. However, the challenge remains to develop a general hypothesis about the role of EVs in physiological homeostasis and pathobiology across kingdoms. While they can act systemically, EVs are often seen to operate locally within a microenvironment. This microenvironment is built as a collection of microunits comprised of cells that interact with each other via EV exchange, EV signaling, EV seeding, and EV disposal. We propose that microunits are part of a larger matrix at the tissue level that collectively communicates with the surrounding environment, including other end‐organ systems. Herein, we offer a working model that encompasses the various facets of EV function in the context of the cell biology and physiology of multicellular organisms.

## 1 INTRODUCTION

Exosomes and microvesicles including apoptotic vesicles or apoptotic bodies are collectively referred to as extracellular vesicles (EVs). Extracellular vesicles in the 50–200 nm range whose origins in the endocytic pathway cannot be confirmed are referred to as small extracellular vesicles or sEVs.^1^ The field of EV biology has been rapidly evolving, becoming a diverse and deep area of study to understand the mechanisms of cellular communication in both homeostatic tissue function and disease.^2^, ^3^ Here, we will summarize important points on these topics and present a hypothesis on the role of EVs in the homeostatic function of microenvironments.

**FIGURE 1:**
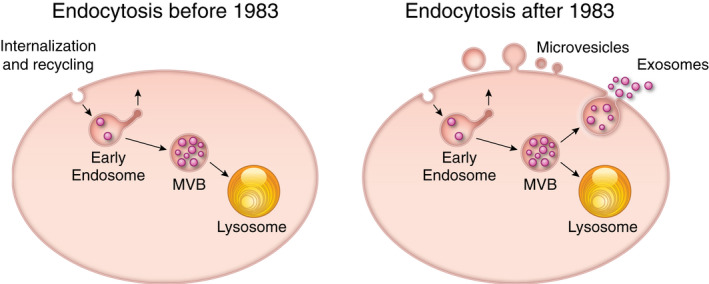
Two papers published in 1983 (Harding, Heuser, and Stahl (1983,^4^) and Pan and Johnstone (1983,^5^) established the basis for a new field of bioscience‐ the biology of exosomes and microvesicles. Starting with Lewis's description of pinocytosis in 1931 and the discovery and characterization of receptor‐mediated endocytosis over the next several decades,^8^ the principle contours of the endocytic pathway (i.e., internalization and recycling) were mostly understood by the early 1980s. Currently, we know that exosome secretion is found in virtually all eukaryotic cells. Moreover, it is more complex than ever could have been imagined after its discovery in 1983, involving elements of the entire endocytic and autophagic network. Additionally, the discovery of a novel vesicle secretion pathway^9^ at the plasma membrane (which we now refer to as microvesicles) has biochemical underpinnings both similar to and uniquely different from exosome biogenesis and secretion. The term extracellular vesicle includes exosomes and microvesicles

The exosome secretion pathway was discovered when two complementary papers, one from Pan and Johnstone and a second from Harding, Heuser, and Stahl,^4^, ^5^, ^6^ appeared virtually simultaneously in the summer of 1983 demonstrating that maturing blood reticulocytes package and jettison their transferrin receptors in small 75‐nm vesicles while leaving other membrane proteins intact. Cliff Harding's article^4^, ^6^ revealed the existence of a new intracellular membrane protein sorting and secretion pathway that had the multivesicular endosome or multivesicular body (MVB) as its locus. The term exosome, initially introduced by Trams et al.^7^ to describe membrane exfoliation was repurposed a few years later to refer to vesicles of endosomal origin (Figure 1). Although not explicitly stated in these discovery papers, it was a commonly held view that the pathway represented a form of selective expulsion or disposal of unwanted or unneeded membrane proteins.

## 2 THE EV CARGO PACKAGING AND THE DISPOSAL HYPOTHESIS

Recently, Michel Vidal^10^ has revisited the concept that exosome biogenesis and secretion represent a physiologic mechanism to rid the cell of unwanted content. To date, many of the examples of selective secretion via exosomes have been reported in the reticulocyte. However, the reticulocyte may not be the best model since they are mostly devoid of lysosomes and thus marginally competent to carry out intercellular degradation. Identifying specific proteins in exosomes that have been packaged and ejected from cells for physiologic reasons is a problem of enormous magnitude with virtually thousands of proteins identified in exosomes from many cellular and tissue sources. Moreover, there is no unifying hypothesis to address the problem experimentally. Proteins might be enzymatically tagged (e.g., ubiquitin or ubiquitin‐like modifiers)^11^ or modified in some other way to promote disposal (e.g., acylation or phosphorylation) or aggregated in lipid domains. Intraluminal vesicles are enriched in cholesterol which may play a role.^12^ Moreover, there are clearly subsets of intraluminal vesicles suggesting multiple biogenetic pathways.^13^

The question of how specific content (i.e., proteins, small RNAs, etc.) is gathered together and packaged into nascent intraluminal vesicles scheduled for secretion is a central question and a key tenant in the EV communication/disposal hypothesis. Intraluminal vesicles within MVBs are formed by multiple assembly pathways.^14^, ^15^ The most well‐characterized mechanism involves elements of the ESCRT family of gene products.^16^ The ESCRT acronym refers to Endosomal Sorting Complexes Required for Transport. VPS4, an ESCRT AAA ATPase, and its partners, have the ability to create a virtual molecular noose on the surface of endosomal membranes such that, as filaments of the noose gather together, the underlying membrane and its content invaginate in a trans direction forming a nascent vesicle. A membrane fusion event then completes the process with the release of an intraluminal vesicle.^17^, ^18^ Moreover, there are ESCRT‐independent pathways of intraluminal vesicle biogenesis that rely on the formation of ceramide, potentially regulated by Rab31.^19^ Some biologically active molecules enter exosomes from the early endocytic pathway, where their transit may require members of the Rab5 or Rab7 families of GTPases. Others arise from the late endosome and may require members of the Rab7 (Rab7a and 7b) or Rab11 (Rab11a and 11b) families. Rab35 along with its activating proteins TBC1D10A and TBC1D10C has been shown to control exosome secretion.^20^ In an extraordinary RNA interference (RNAi) screen, Thery and colleagues demonstrated that Rab27a and Rab27b and their regulatory co‐factors control the secretion of exosomes.^21^

Recently, Schekman and colleagues^22^ reported the first in vitro reconstitution of exosome assembly in a cell‐free system, where they suggested that ubiquitination may play a role in the packaging of exosomal content. Zimmermann and colleagues^23^ have identified other variations of ESCRT‐dependent assembly involving phospholipase D2, syntenin, and syndecan. A second pathway called the LDELS pathway (LC3‐dependent EV loading and secretion) has been recently described, which uses the autophagy initiator LC3.^24^ RNA‐binding proteins are recruited by LC3, followed by loading into intraluminal vesicles, opening up the possibility for selective packaging of miRNA and other biologically active RNAs. In the novel LDELS pathway, vesicle biogenesis is achieved by the generation of ceramide *supra vide*, a membrane‐bending lipid, corroborating the functional connection between exosome biogenesis and the autophagy pathway.^25^ The fact that some MVBs deliver content to the lysosome for degradation while others fuse with the plasma membrane suggests that there may be multiple types of MVBs, perhaps with functions yet to be discovered; in fact, a number of studies confirm these suspicions.^13^, ^26^

Additionally, work with polarized cells indicates that secretion of intraluminal vesicles from the apical surface differs from those released from the basolateral surface, further suggesting that different kinds of MVBs exist.^27^, ^28^ In tandem, as EVs also emerge from the cell surface, with sizes that can range from 100 to 1000 nm, unique regulatory mechanisms controlling their release from the cell are likely involved.^29^ Microvesicles (MVs) for instance, which are on the large end of the size scale, are formed and known to be dispatched by several mechanisms—some requiring Arf6, while others, called ARMMs, involve elements of the ESCRT family. The diverse pathways involved in the packaging and release of EVs are just beginning to be fully appreciated and are likely to help introduce new distinctions between EV subtypes moving forward.

As part of these endeavors, analysis of EV content has been the subject of many studies and an entire compendium (i.e., vesiclepedia) is available for inspection. (Reader be warned, vesicles cataloged in this resource were isolated by many different techniques making comparisons imprecise). Studies with EVs isolated from urine may be particularly informative.^30^, ^31^, ^32^ Urinary EVs are derived from the epithelial cells of the kidney and bladder. Two recent papers^33^, ^34^ may offer deeper clues about the EV disposal hypothesis. Proteomic analysis of urine‐derived EVs indicates the presence of a plethora of ubiquitinated proteins with multiple ubiquitin linkages. One conclusion from the work is that de‐ubiquitination is not required for export. Ubiquitination and de‐ubiquitination are key elements of the recruitment of proteins marked for degradation in the proteasome or via the recruitment of ESCRT complexes for delivery to MVBs. Why does one find ubiquitinated proteins in urine‐derived EVs? These observations suggest that the capture of selected proteins targeted for disposal by incorporation into EVs and secreted may be ubiquitin dependent. The second set of observations with urinary EVs, equally remarkable, is the finding that they are enriched in mannosylated glycopeptides. Why are mannosylated glycopeptides found in higher abundance than complex chains in urinary EVs? Most glycoproteins bear N‐linked chains of the complex type having been processed in the Golgi apparatus during biosynthesis. High mannose chains are in the minority. Mannosylated glycopeptides found in exosomes may be associated with newly synthesized glycoproteins in the ER‐‐proteins that, because they are misfolded or improperly folded, are destined for transport to the cytosol via ERAD (ER‐associated degradation) or ERLAD (ER to lysosome‐associated degradation). In ERAD, translocated misfolded proteins are thought to be degraded by proteasome capture and digestion. However, with ERLAD, misfolded proteins are clustered in the ER by ER‐phagy receptors such as FAM134b, followed by vesiculation.^35^ The newly formed vesicles containing aggregated and misfolded mannosylated glycoproteins may fuse directly with lysosomes. Alternatively, vesicles derived from the ERLAD pathway may be diverted to autophagosomes or to MVBs in preparation for secretion as EVs. This could account for the high content of mannosylated glycopeptides in urinary EVs. Another class of proteins found in EVs are cytosolic‐derived aggregated proteins such as alpha‐synuclein (found in plasma‐derived EVs). Alpha‐synuclein packaged in EVs may be involved in the spread of certain neurological diseases such as Parkinson's disease. Understanding the mechanisms of unfolded and aggregated protein export in exosomes and microvesicles may open new therapeutic opportunities for a score of neurodegenerative diseases.

Finally, the disposal of misfolded proteins in eukaryotic cells may have its roots in the ejection of misfolded proteins by bacteria and archaea.^36^ Gram‐negative bacteria have been shown to vesiculate during stress and to eject misfolded proteins. It is conceivable that a universal mechanism is operative in healthy cells allowing them to shed unwanted or misfolded/aggregated proteins.

## 3 THE EV COMMUNICATION HYPOTHESIS

**FIGURE 2:**
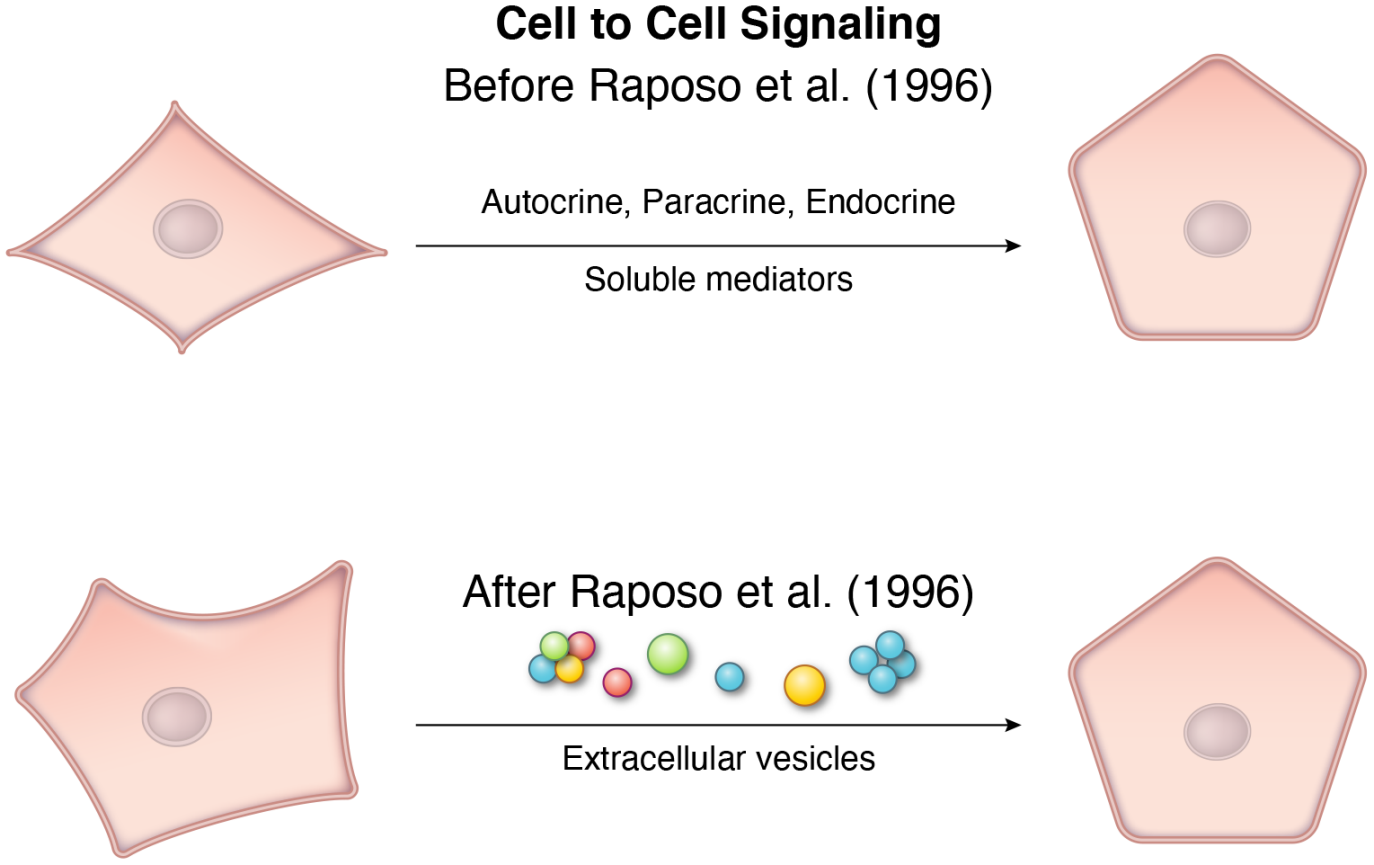
The concept of EVs as conveyors of the cell to cell communication. The concept that cells can communicate by packaging informational content in EVs was first advanced by Raposo et al.^37^ using antigen processing and presentation as a model. Prior to that communication was known to be mediated exclusively by soluble factors in the form of paracrine, autocrine, and endocrine signals. Among the questions that need to be resolved is whether EV signaling is mediated or carried by a single vesicle or whether a form of quorum signaling is involved including whether vesicles are clustered. Exosomes may be clustered by the exosome linker protein tetherin.^38^ Clustering may be true of microvesicles as well. Clusters may be homogeneous or a heterogeneous mixture of vesicles where each vesicle in the cluster is necessary but not sufficient for signal transduction. Subsequent work by many groups, especially after the discovery of EVs as carriers of RNA, has amply confirmed that EV signaling is a form of the cell to cell communication that may also include other members of the biological kingdom, archaea, bacteria, and plants

A decade after Pan and Johnstone and Harding, Heuser and Stahl, a new concept was introduced by Graca Raposo and colleagues,^37^ reporting that exosomes operate as signaling entities able to mediate communication between cells. The exosome‐signaling hypothesis was, thus, established via a paper, now classic, that exosomes secreted by an antigen‐processing cell can present antigen to a T cell. Using immunogold cryo‐electron microscopy, Raposo et.al demonstrated that biologically active exosomes, bearing MHC class 2 molecules, are secreted by antigen‐presenting cells.^37^ This paper opened up EV research to a much broader audience and interest across the biomedical research world began to intensify (Figure 2). Are individual exosomes harboring MHC antigens necessary and sufficient to deliver information to an acceptor cell? Or, is signaling managed by a quorum or threshold effect similar to quorum sensing in bacteria? These are fundamental questions that may only be resolved with the isolation and characterization of individual exosomes. The technology is not available yet, nor on the horizon, to examine the “omics” or comprehensive composition of individual exosomes—indirect methods will need to be developed to analyze donor EVs and the recipient cell responses.

An additional bolster to EV research was provided by the work of two groups headed by Mariusz Ratajczak^39^ and Jan Lotvall,^40^ who separately demonstrated that EVs bearing mRNA and miRNA could mediate the horizontal transfer of biologically active nucleic acids from one cell to another. These observations confirmed the concept of EV‐based signaling and opened up new conversations about the physiological and potential therapeutic role of EVs, while highlighting the need for more fundamental work on the mechanisms of RNA packaging.

**FIGURE 3:**
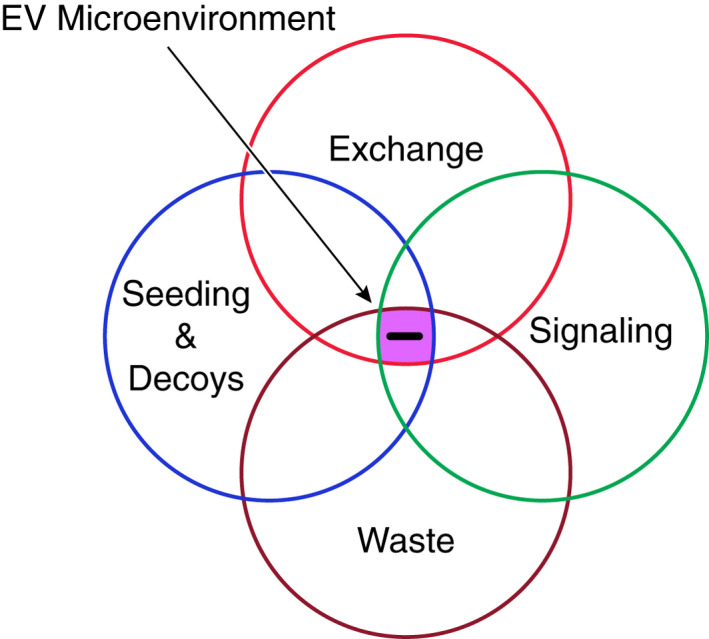
The microenvironment view of EV function. Homeostasis (a term proposed by W.B. Cannon^41^, ^42^ in the early part of the 20th century) is the aggregate of those forces within organisms that maintain the constancy of the internal environment—the milieu interieur—a concept introduced by Claude Bernard in the 19th century. The role of EVs in homeostasis might be best viewed in the context of local tissue environments or microenvironments. Here, the collective action of several overlapping “EV functional units” including EV exchange (between and among cells), EV seeding of the microenvironment^43^, ^44^ refers to the secretion of EVs that are enzymatically endowed resulting in the level of specific metabolites or signaling molecules in the microenvironment or the deployment of “decoys” that could serve as alternative targets for invading pathogens,^45^ EV signaling (the specific targeting of signaling molecules (e.g., transfer of antigens associated with MHC class II molecules^37^ or the transfer of MiR690 to insulin‐sensitive tissues to improve insulin sensitivity^46^) between cells whether within tissues or between tissues) and EV disposal (waste), the active disposal of unneeded or defective proteins, come together to support and sustain the local environment. Homeostasis is achieved at the crossroads of these functional units

Over the last decade, the EV field has flourished, with many new reports touching nearly every field of study under the biological umbrella. One of the challenges before the EV research community now is to develop an understanding of EV biology as a system—a system of overlapping and increasingly complex pathways, including the cell biology of biogenesis and secretion and from a physiological point of view, exchange, seeding, and signaling (see Figure 3) allowing cells to partner with other cells and their microenvironment.

## 4 EXPERIMENTAL MODELS

To date, thousands of papers have been written about EVs, a significant fraction of which are reviews (reflective of the early stage of this rapidly developing but still largely undefined field). In spite of all of the great progress, we still do not have a precise handle on the physiological function of EVs that informs our understanding of human homeostasis. For therapeutic applications, human physiology and disease models will be critically important, but human research will be rather limiting for mechanism discovery because it will be difficult to interrogate these models experimentally for both practical and ethical reasons. The recent introduction of spheroids and organoids as a research tool offers an entirely new line of investigation that may aid our understanding of EVs in human physiology and disease.^47^ Spheroids, from human tumors and various tissues, along with organoids, derived from stem cells that are allowed to grow on a matrix, have now been established for the brain, liver, kidney, and adipose tissue (AT) where the assembled cell mass can be used to study the interaction of different cell types. A case in point is a recent study by Cline and colleagues on Rett syndrome, a devastating brain disorder linked to the loss of the methyl‐CpG‐binding protein 2 (MECP2) that leads to delayed brain development.^48^ Intercellular connections via neural circuits can be followed within organoids derived from MECP2‐deficient progenitors. Depressed signaling via neural circuitry was detected in the MECP2 deficient cultures. Treatment of MECP2‐deficient organoids with exosomes derived from normal MECP2 sufficient organoids led to a partial normalization of neural circuitry. While still early, these kinds of studies open up a number of experimental approaches to delineate the function of EVs, not only in developmental neurobiology, but also throughout human biology.

A second very promising approach to develop an understanding of the homeostatic role of EVs and to solve the molecular mechanisms that underwrite all of the abovementioned units of function (namely: exchange, seeding, and signaling), is the use of model organisms. A number of excellent animal models have been used for discovery purposes and to understand the molecular cell biology and physiology of EVs in multicellular organisms—the mouse, fish, fly, and worm among others. One of the best animal models for the study of developmental processes is the zebrafish.^49^, ^50^, ^51^ Over the past few decades, the zebrafish has become a “go to” model organism for many researchers, being a genetically accessible, vertebrate model that is easy to care for and maintain. Zebrafish are also translucent as embryos, making them ideal for longitudinal imaging studies across the whole of development. Another key feature of zebrafish is that they are more similar to humans than the fly or worm. The doubling of the fish genome 350M years ago, along with segmental duplications, led to diversification and expansion of the fish genome making it more relevant to humans—with over 70% of protein‐coding genes having human orthologs, and over 80% of human disease‐causing genes being represented. Zebrafish expressing CD63‐pHluorin or membrane‐tethered fluorphors have been developed to label and track EVs in vivo.^49^, ^50^, ^51^ The strength of the fish model is the ability to watch EV trafficking and mobilization in a living vertebrate system. Through the use of these new experimental tools, the study of EV biology in respect to organismal homeostasis can be expected to continue apace.

Lastly, significant progress has been made in the plant world of EV research such that we can now confirm that an entirely new field of investigation is taking shape. Plant cells produce EVs that are engaged in the daily activities of communication and exchange including defense against pathogens—a “heads up” of things to come.^52^ Just as EVs in animal cells will influence the practice of medicine, EVs in plant cells may influence the future of agriculture.

In sum, delineating the homeostatic effects of EVs in the microenvironment and beyond is likely to change our understanding of physiology and pathophysiology in plants and animals and has an important, if not paradigm shifting, impact on diagnosis and therapeutics. However, much is yet to be done to understand the basic physiology that underlies EV usage in tissue and organismal homeostasis.

## 5 ELEMENTS OF A HOMEOSTATIC THEORY

EVs are omnipresent in biological fluids and include apoptotic vesicles, large and small microvesicles, and exosomes. It is the latter of these two, microvesicles and exosomes, that appear to be involved, perhaps exclusively, in EV homeostatic regulatory events. EVs, which are released by virtually all cells, are diverse with respect to size and content. From all of this complexity, how do we evolve a general understanding of the physiological importance of EVs? Testable theories of EV function will need to make sense of a plethora of observations—multiple mechanisms of EV biogenesis, regulated and non‐regulated secretion, vesicle transport and targeting (both local and long distances), intracellular delivery to target cells and their microenvironments, and finally function, both in physiological and pathological contexts.

How can we parse the roles of various EV activities in the context of a functional unit? A functional unit here is defined as the selective transfer of content from one cell to another or from one cell to a specific extracellular site (e.g., extracellular matrix). Functional units might include the following: (i) cellular housekeeping, (ii) exchange and commingling between cells and tissues, (iii) seeding the microenvironment (including decoy activities^45^), and (iv) signaling (Figure 3).

Cellular housekeeping refers to the specific packaging and shedding of biologically active molecules where a physiological need is satisfied.^10^ Examples include the jettisoning of transferrin receptors or aquaporin molecules from maturing reticulocytes or the dumping of phospho‐MLKL from cells undergoing necroptosis.^53^ Exchange refers to the transport of molecules from one cell to another that are (i) necessary for survival, (ii) provide homeostatic stability (e.g., the ability to synthesize NAD^+^ ^54^), or (iii) protection during stress (e.g., heat shock proteins).^55^ Seeding the microenvironment provides a mechanism in multicellular organisms to support a physiological function such as extracellular queuing of signals during cell migration (e.g., chemotaxis^44^), modifying the microenvironment by delivering needed metabolic substrates,^43^ or by secretion of decoys^45^ whose function is to protect the organism from invading pathogens. Lastly, EVs offer a mechanism to connect cells by presenting specific signaling instructions, where the generated EVs target their cognate partners with high specificity. Prime examples include antigen presentation, where recent work shows the specific transfer of a set of miRNAs that regulate T cells,^56^ the choreography of tissue developmental pathways, in which WNT is secreted via exosomes and targets cells with frizzled/WNT receptors^57^ and the secretion of EVs from macrophages that enhance insulin signaling in a variety of cells.^46^ In reproductive biology, the transfer of human‐specific miRNAs from the placenta to endothelial cells to protect against viral infections serves both as a novel signaling mechanism and as a harbinger of uncharted territory, human‐specific EV signaling, and exchange pathways.^58^ In physiology, this concept holds true; signaling within a tissue microenvironment can have profound effects on whole‐body health or pathology. Prime examples of this are adipose tissue and the cardiovascular system where coordination between multiple cell types in the tissue is required to maintain homeostasis. In both these examples, dysregulation of intracellular communication can have significant adverse effects on the whole organism, suggesting the role of EVs in communicating across microenvironments.

## 6 EVS IN THE MICROENVIRONMENT—EXEMPLARS

The adipose tissue microenvironment, the cardiovascular microenvironment, the skin and wound healing, the brain microenvironment, and the tumor microenvironment among many others, are under extensive study from both a physiological and pathobiological perspective.

### 6.1 EVs in the adipose tissue microenvironment

The AT is an example of a tissue microenvironment strongly influenced by EV signaling. The AT plays an essential role in systemic adaption to nutrient excess or deprivation by receiving signals about the energetic demands of the system and modulating the availability of energetic substrates to cells in all organs. The healthy expansion or contraction of white AT relies on an appropriate, coordinated response of adipocytes, endothelial cells, immune cells, and fibroblasts. The adipocyte is at the center of this concerted response through secretion of pro‐ and anti‐inflammatory cytokines, endothelial growth factors, extracellular matrix proteins, adipokines, and EVs to regulate the function of stromal vascular cells. In recent years, we have learned that, in mice, there is a significant exchange of EV material between cells in the adipose tissue.^59^ Given the complexity of AT EV cargo, these transfer events likely represent an expansive and robust signaling network. For this reason, EV‐mediated signaling has entered the forefront of investigations into intercellular communication in adipose tissue. Recent work has focused on pathological signaling between cells in AT in the context of obesity, type 2 diabetes, and cancer; however, little is known about EV signaling under normal physiological conditions. The biogenesis and release of EVs are enhanced in response to varied forms of stress: inflammation, oxidative stress, osmotic stress, fatty acids, low pH, and nutrient deficiency. This suggests that EVs likely play a significant role in acute adaptation to stress to maintain homeostasis in all tissues, including AT. EVs have been shown to modulate key processes in AT: cell fate, wound healing, and angiogenesis.

Adipocyte‐derived EVs seem to be the dominant source of EVs in the AT. Adipocyte EVs are taken up by the major cell populations in adipose tissue: immune cells, stem cells, and endothelial cells^59^; however, little is known about the specific signals that are communicated via these EVs. The most well‐studied axis of AT EV‐mediated signaling is between adipocytes and macrophages. This interaction has been studied mainly in the pathological state of obesity where adipose tissue becomes highly inflamed.^60^ However, adipocyte‐to‐macrophage crosstalk is essential for functional AT under healthy physiological conditions. Throughout development, bone marrow‐derived monocytes populate all tissues. Upon interaction with the unique microenvironment of each tissue, monocytes are stimulated to differentiate into macrophages with tissue‐specific properties.^61^ This is the result of reciprocal interactions with cells in the tissue and contact with the extracellular matrix and interstitial fluid.^61^ Recent studies have demonstrated that adipocyte EVs may be sufficient to instruct monocytes to take on characteristics that are specific to AT macrophages as they differentiate.^62^, ^63^ This is at least partially due to the EV‐mediated transfer of whole lipid droplets from adipocytes to macrophages, which stimulate macrophage lipid accumulation, multinucleation, and increased lysosome content, all characteristics of adipose tissue macrophages.^62^ Furthermore, once macrophages are differentiated, they exist on a continuum of polarization from an M1‐like pro‐inflammatory phenotype to an M2‐like anti‐inflammatory, housekeeping phenotype.^60^ Adipocyte EVs actively influence this polarization. EVs released from adipocytes in the stress state of overnutrition carry miR‐155, and sonic Hedgehog to promote M1‐like macrophage polarization and miR‐34a to inhibit an M2‐like phenotype.^64^, ^65^, ^66^ In contrast, pro‐inflammatory AT macrophage EVs did not affect preadipocyte differentiation into mature adipocytes.^67^ This may be an example of how EVs in the tissue can fulfill a “seeding” function to provide queues in the microenvironment to fine‐tune macrophage recruitment and/or function.

Macrophages also reciprocate EV‐mediated signals to adipocytes. In obesity, AT macrophages that are polarized into an inflammatory, M1‐like phenotype shed EVs that promote inflammation and insulin resistance in adipocytes.^67^, ^68^, ^69^ These macrophage EVs can also enter circulation and have a similar effect system wide by inducing insulin resistance in the liver and muscle as well.^68^ In contrast, M2‐polarized macrophage EVs have the opposite effect on adipocyte function: improved insulin signaling in AT, muscle, and liver, resulting in improved whole body metabolism.^46^ Therefore, the adipocyte EVs strongly influence macrophage activation, which, in turn, modulates the function of adipocytes themselves.

Like macrophages, adipocyte EVs may also dictate the identity of other cells in AT. Adipocyte EVs have been shown to promote differentiation of preadipocytes into mature adipocytes in both homeostatic and pathologic conditions. Like macrophages, adipocyte precursors also exist in functionally distinct populations: fibro‐inflammatory progenitors (FIPs) that lack adipogenic potential and are profibrogenic and pro‐inflammatory and adipocyte precursor cells (APCs), which are highly adipogenic.^70^ This heterogeneity of the pre‐adipocyte population is a recent discovery; thus, no studies have investigated the potential role of adipocyte EVs in the fate of FIPs or APCs. However, what we know about the function of adipocyte EVs in macrophage polarization makes a role in precursor polarization plausible. This also suggests the potential paradigm that adipocyte EVs may be critical for the establishment of the adipose tissue microenvironment through both seeding and signaling functions.

Adipocyte EVs also serve an important cell autonomous housekeeping task of removing unwanted or toxic materials from the cell. For example, mitochondrial dysfunction in adipocytes induces the packaging of fragmented, damaged mitochondria into EVs, which are then extruded from the cell.^71^ In addition, EVs are also thought to protect the adipocyte by carrying ceramides out of the cell, a potentially toxic lipid species.^72^ Adipocyte EVs are predominantly taken up by resident macrophages.^59^, ^62^ Therefore, once EVs are shed from the adipocyte, it is then likely up to the specialized AT macrophages to take up and degrade this unwanted material. This concept of EVs mediating the “out‐sourcing” of waste disposal has also been reported in the stem cell niche where mesenchymal stem cells release damaged mitochondria to be degraded by macrophages.^73^

**FIGURE 4:**
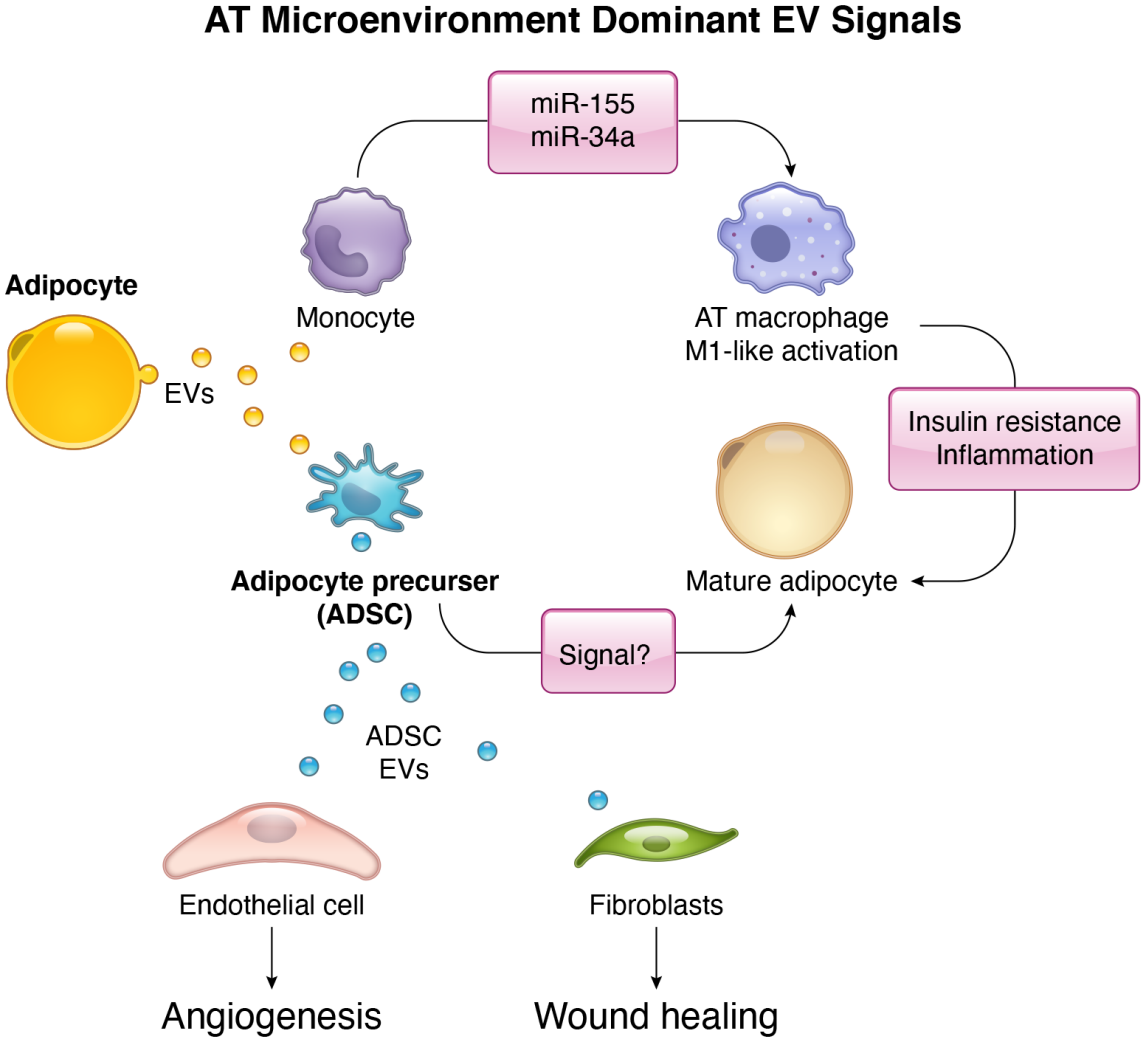
Dominant signals in the AT microenvironment. Adipocytes and ADSCs are two cell types in the AT tissue that robustly signals via EVs. Adipocyte EVs can promote preadipocyte differentiation into mature adipocytes. Additionally, adipocyte EVs also stimulate the differentiation of monocytes into macrophages and promote an M1‐like macrophage polarization. In turn, the M1‐like macrophages release EVs that induce insulin resistance and inflammation in adipocytes. ADSC EVs stimulate wound healing by promoting angiogenesis, fibroblast proliferation, re‐epithelialization, and collagen deposition

Adipose‐derived stem cells (ADSCs) are by far the most well‐studied source EVs in adipose tissue and have a strong signaling function. These cells are self‐renewing and multipotent but frequently referred to as adipocyte progenitors. The major function of ADSCs EVs is stimulating wound healing. This function is important in large adipose depots in the instance of soft‐tissue trauma or chronic obesity where the tissue is damaged by mechanical force, or metabolic and inflammatory processes, respectively. In a housekeeping context, dermal adipose tissue plays an essential role in cutaneous wound healing and hair growth.^74^, ^75^ The dermal adipose tissue is a thin layer of adipose tissue under the reticular dermis and completely distinct anatomically and developmentally from subcutaneous adipose tissue.^76^ Interestingly, the concentration of EVs in the dermal interstitial fluid is about 12 times higher than that of serum in humans and rats, suggesting this is a robust site of EV function.^77^ In the context of soft‐tissue injury, both sEVs (small extracellular vesicles approximating the size of exosomes but not confirmed to have a MVB source) and microvesicles from ADSCs promote wound healing by enhancing the proliferation and migration of endothelial cells and fibroblasts, promoting collagen deposition to strengthen the injured area, and preventing apoptosis.^78^, ^79^, ^80^, ^81^ This may be primarily true during the early stages of healing as ADSC EVs may inhibit collagen synthesis during later stages to prevent fibrosis.^78^ This results in increased re‐epithelialization, collagen deposition, and neovascularization to accelerate wound closure in vivo.^78^, ^80^ ADSC EVs have been shown to modulate wound healing via activation of AKT, ERK, and Wnt/β‐catenin signaling in receiving cells.^80^, ^81^ In addition, ADSC EVs carry vimentin, a cytoskeletal protein that forms a protective cage‐like mesh around the nucleus and contributes to the mechanical strength of the cell.^82^ EV‐associated vimentin has been shown to contribute to wound healing by inhibiting osmotic stress‐induced apoptosis of fibroblasts, a stress that is caused by epithelial breaches.^82^ Interestingly, sEVs from ADSCs home to soft‐tissue wounds following intravenous injection and enhance wound closure.^78^ In this way, ADSC sEVs may act both locally, in the dermis, and systemically (Figure 4).

ADSC EVs have also been shown to have strong angiogenesis‐stimulating potential, a property that contributes to their wound healing function.^80^, ^83^ The vascular network of AT is dense and highly dynamic. 3D volume fluorescence imaging has revealed that the vascular density of AT can change dramatically under several physiological and pathophysiological conditions such as cold exposure and obesity.^84^ This suggests that there is active communication between cells in the tissue to regulate neovascularization and blood vessel regression under specific conditions. It is clear that ADSCs strongly impact the angiogenic capacity of AT^85^; however, the importance of ADSC EVs in this process is not well studied. ADSC EV‐stimulated angiogenesis has been demonstrated in the fields of regenerative and cosmetic medicine, where the angiogenic property of these EVs was exploited to support viable tissue grafts, and to stimulate tissue regeneration after ischemic damage.^83^, ^86^ Based on these findings, it is likely that ADSC EVs contribute to the endogenous angiogenic properties of AT. For example, Han et al. demonstrated that EVs isolated from human ADSCs under hypoxic conditions enhanced the proliferation, migration, and tube formation of human umbilical vein endothelial cells (HUVECs^87^;). In addition to ADSC EVs, adipocyte‐derived EVs may be an important modulator of endothelial cell (EC) function. In a pro‐inflammatory AT environment, like that of obesity, adipocytes release EVs that upregulate cell adhesion proteins in HUVECs such as E‐selectin, P‐selectin, and VE‐cadherin.^88^ This results in significantly more leukocyte attachment to HUVECs.^88^ Furthermore, EVs from insulin‐resistant adipocytes promote pathological forms of angiogenesis.^89^ Outside of disease states, adipocytes and ECs robustly exchange EVs in healthy mouse AT.^59^ This suggests that EV‐mediated crosstalk may be an important modulator of angiogenesis, particularly between adipocytes, ADSCs, and ECs.

### 6.2 EVs in cardiovascular homeostasis

Conversely, endothelial cells, the cells of the cardiovascular system directly in contact with blood cells and hemodynamics, have been shown to secrete their own EVs with similar physical characteristics to those reported for other cells and tissues. Crosstalk between tissues, such as that between AT and endothelial cells, reveals how large, complex, and multicellular microenvironments of EV transfer can be to dictate homeostatic events at the level of an organism. Within the cardiovascular system more specifically, endothelial cells receive both autonomous and non‐autonomous signals via EVs to dictate their function.^90^, ^91^ Since the early papers describing EV involvement in endothelial cell biology in the 1990s and early 2000s,^92^, ^93^, ^94^ significant interest has been paid to understating how and where EVs are generated, how EVs are targeted, and what content EVs carry to alter vascular homeostasis.

Like EVs isolated from most other tissues, EVs altering endothelial function have been shown to carry a wide range of contents including miRNAs, mRNA, signaling receptors, transcription factors, and chemokines/growth factors to directly influence cellular behavior. The contents of individual EVs seem to represent the composition and the activation state of the cell type they derived from and seem to largely be taken up by the endothelium through endocytic pathways.^90^, ^95^ In addition to EVs derived from ADSCs (as discussed above) and endothelial cells themself, there are multiple other non‐autonomous cellular sources of EVs that have been shown to alter cardiovascular function—including mesenchymal stem cells, stromal cells, the brain parenchyma, platelets, leukocytes, erythrocytes, and monocytes/macrophages.^90^, ^91^

Taking the process of angiogenesis as an example, endothelial autonomous and non‐autonomous EV uptake has been shown to promote angiogenesis via transfer of microRNAs such as miRNA 31, 125a, 126, 150, 214, and 296.^96^, ^97^, ^98^, ^99^, ^100^, ^101^ Further proteins and transcription factors such as Stat3/5, NF‐kB, VEGF, FGF, S1P, SCF, and PDGF have been shown to be present in EVs and functional to drive differences in angiogenic potential.^91^, ^102^, ^103^, ^104^, ^105^, ^106^, ^107^, ^108^ EV transfer seems to ultimately lead to signaling activation through PI3K, Erk1/2, Wnt/beta‐catenin, and NF‐kB activation in receiving endothelial cells to promote angiogenesis and/or endothelial cell motility.^102^, ^103^, ^106^, ^107^, ^108^, ^109^ Interestingly, anti‐angiogenic effects have also been noted from EV transfer. For instance, if EVs are collected under conditions of cellular stress or inflammation, they often continue to carry these signatures and transfer stress signals to receiving cells. From this concept, the anti‐angiogenic properties of EVs have been in part linked to altered EV uptake via CD36‐dependent mechanisms and induction of oxidative stress.^110^, ^111^, ^112^

The implications of these types of interactions are far reaching for the cardiovascular system, which is obviously highly connected through its miles of blood vessels. Though dramatically understudied for their role in the development and homeostatic events, the concept of EVs as a cell‐to‐cell communication strategy to coordinate vascular response to hypoxia or nutrient insufficiency systemically is compelling. EVs released from endothelial cells and mesenchymal stem cells have been shown to be released into circulation in response to hypoxic events, such as myocardial infarction, to target cardiac cells for repair, prevent apoptosis, and promote vascular regeneration. Within these contexts, there is signaling from cardiovascular cells to immune cells, such as macrophages, and vice versa. While the majority of functional studies have been largely carried out utilizing EVs collected from isolated cell populations in culture that are then re‐administered in vivo, the concept holds that EVs in this context have been shown to reduce myocardial infarct size, reduce scaring/fibrosis, promote neo‐angiogenesis, and improve cardiac function (a subset of this work is referenced here^51^, ^100^, ^102^, ^103^, ^106^, ^107^, ^108^, ^109^, ^113^, ^114^, ^115^, ^116^, ^117^, ^118^, ^119^). One of the next major undertakings will be understanding the endogenous transfer of EVs in their native environment, and how this signaling impacts cardiovascular development and homeostasis under non‐pathologic conditions.

The microenvironments described above represent a minor sampling of the many physiologically important microenvironments that exist across the plant and animal kingdoms where EVs may play key roles in signaling, exchange, seeding, and waste removal.

## 7 THE MOLECULAR LANGUAGE OF EV COMMUNICATION

Given the growing knowledge surrounding the interconnection of microenvironments via EV transferred signals, the next big challenge is understanding the question—what is the molecular language that underwrites EV communication and exchange? EV communications, when viewed as a system, will be best understood by establishing a set of fundamental principles. First, all vesicles involved in homeostasis are endowed with certain cognate recognition molecules on their surfaces that oversee their targeting and fate. Second, recipient cells possess the appropriate receptor sites to accommodate EV binding (and uptake). Third, the packaging of appropriate content in newly formed vesicles is concomitant with the assembly of cognate recognition molecules during vesicle biogenesis. The specificity encoded in EV surface recognition molecules can be broad or narrow. At one end of the spectrum, certain EVs could have a low level of specificity that would allow them to deliver their content within specific tissues or across tissues. Evidence to date suggests that integrins may be involved in the tissue‐specific targeting of EVs.^120^ Even vesicles generated during housekeeping events may have targeting information that would enhance their disposal via the reticuloendothelial system. The second level of specificity might include information that would allow EVs to target extracellular matrix (ECM) molecules that would promote their function in a microenvironment. The highest level of specificity might be reserved for molecular cues similar to that found in hormone or cytokine‐based targeting. For example, EVs decorated with WNT are highly specific in delivering information content via WNT receptors to enhance a developmental pathway. Add to the complexity, recognition of EVs by cell targets could be combinatorial where multiple molecular elements or cues are involved. Newly formed EVs may assemble as clusters (see Figure 2), possibly connected by an exosomal tether called tetherin.^38^ EV clusters may offer a way to improve both specificity and payload content in EV delivery. Particularly relevant to the internalization of EVs by target cells is the family of Rab GTPases that regulate membrane trafficking along the endocytosis pathway. The human genome has over 60 Rab GTPases, as does the zebrafish.^121^ Model organisms lower in the phylogenetic scale have fewer Rab genes/isoforms. For example, the GTPase Rab5, the gatekeeper for endocytosis, exists as a single copy gene in worm and fly but is present as at least four separate paralogs in fish and three in humans (Rab5a, Rab5b, and Rab5c) giving rise to a more complicated endocytic network than is present in lower order organisms.^122^, ^123^ Elucidation of these navigational cues, how they are encoded, and how they operate to effect targeting and delivery of EVs is essential, both to our understanding of EV homeostasis in multicellular organisms and to understanding the impact of EVs on human physiology and pathophysiology.

## 8 A GENERAL HYPOTHESIS FOR EV‐MEDIATED HOMEOSTASIS

A general hypothesis for EV‐mediated homeostasis in metazoans would consist of two components—a long‐range component and a short‐range component, the former consisting of exchange and signaling activities and the latter of exchange, signaling, seeding, and disposal activities. The latter is the microenvironment.

**FIGURE 5:**
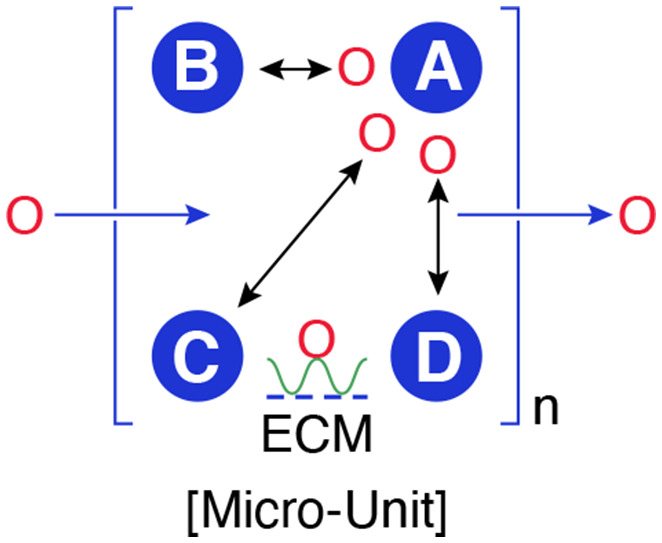
Model for the regulation of the local microenvironment by EVs. The microenvironment (n) is composed of a collection of microunits (shown by brackets). Within a microunit, cells A, B, C, and D interact with each other by secreting and responding to EVs (O). There may be many microunits (n) within a given tissue. The extracellular matrix (ECM) may play a role by sequestering EVs that are either “enzymatically endowed”^43^ or enriched in growth or regulatory factors to promote equilibrium. There may be a dominant cell (e.g., A) that orchestrates the interactions among the others such as adipocytes appear to do in adipose tissue. Microenvironments may respond to EVs from outside their niche or secrete EVs that target distant tissues (e.g., adipose tissue releasing EVs that target liver or muscle). Sites of infection or disease (e.g., cancer) could be evaluated at the microenvironmental level where the same EV‐dependent communication occurs

As shown in Figure 3, communications among local groupings of cells and their microenvironments, depending on the ambient physiological conditions, are carried out by EVs secreted by one set of cells (e.g., resident macrophages) and responded to by the other sets of cells. The extracellular matrix is a potential target for EVs that help maintain the local extracellular milieu. The microenvironment as described in Figure 5 is composed of a collection of microunits, defined as a collection of cells that communicate with each other. Adipose tissue may offer the best model to understand the role of multiple cells in a microunit. The adipose tissue microunit appears to consist of four different cell types—endothelial cells, macrophages, stem cells, and adipocytes, where they coordinate homeostasis by secreting and responding to EVs.^124^ Collectively, they release EVs that influence the overall metabolism and well‐being of the organism. Other microenvironments that may offer windows into the role of EVs in homeostasis, local and long range, include skin, brain, and lung among others.

What are the first steps toward developing a general hypothesis for the role of EVs in homeostasis at the mechanistic level? The fundamental unit of EV biology in metazoans is the microenvironment and the working hypothesis is that multiple microunits in a particular tissue operate both separately and together as part of a larger interacting matrix to promote local homeostasis and by virtue of that equilibrium, provide messaging to other control systems within an organism (e.g., endocrine control). Looking to the future, the immediate goal would be to identify the key EVs from multiple cell sources within a given microenvironment and to characterize their content, the regulation of their secretion, and to use this information to begin to develop a map, not unlike the metabolic maps of yesteryear. The discovery phase must precede the deductive phase. Technology will have to catch up with these aspirations. In the meanwhile, a key component of the discovery process is to evaluate the impact of pathology on the exchange, signaling, seeding, and disposal within the microenvironment.

## ACKNOWLEDGMENTS

The authors thank Graça Raposo and Guillaume van Niel for their helpful comments and sage insight during the development of this manuscript. Clair Crew is supported by NIH R00 DK122019. Amber Stratman is supported by NIH R35 GM137976 and a Cancer Research Foundation Young Investigator Award.

## AUTHOR CONTRIBUTIONS

A. N. Strathman, C. Crew, and P. D. Stahl contributed equally in the writing of the manuscript.

## CREDIT STATEMENT

**Amber N. Stratman**: Writing, review and editing. **Clair Crewe**: Writing, review and editing. **Philip D. Stahl**: Writing, review and editing.
